# Accuracy and clinical safety of guided root end resection with a trephine: a case series

**DOI:** 10.1186/s13005-019-0214-8

**Published:** 2019-12-21

**Authors:** Márk Antal, Eszter Nagy, Gábor Braunitzer, Márk Fráter, József Piffkó

**Affiliations:** 1 Department of Operative and Esthetic Dentistry, University of Szeged, Faculty of Dentistry, Szeged, Hungary; 2 dicomLAB Dental, Ltd., Szeged, Hungary; 3 Department of Oral and Maxillofacial Surgery, University of Szeged, Faculty of Medicine, Szeged, Hungary

**Keywords:** Surgical template, Endodontic microsurgery, Trephine, Apicectomy, Computer guided minimally invasive endosurgery

## Abstract

**Background:**

Root-end resection is an endodontic surgical intervention that requires high precision so that all ramifications and lateral canals so as infected tissues are eliminated. An exploratory study was conducted to justify the clinical safety and accuracy of guided root-end resection with a trephine.

**Methods:**

Fourteen root-end resections were performed in 11 patients. With the aid of computer tomography and rapid prototyping a stereolithographically fabricated, tooth-supported surgical template was used to guide trephinations. Surgery was performed using the printed surgical stent and a trephine was used not only for the osteotomy but for the root end resection as well.

**Results:**

The root end was successfully and completely resected by the trephine in all cases. No intraoperative complications were observed in any of the cases, and the patients were free of symptoms indicating recurrence or complications at the 6-month follow-up. The median angular deviation of the trephination was 3.95° (95% CI: 2.1–5.9), comparable to the angular deviation of guided implant surgery. The mean apex removal error (ARE) was 0.19 mm (95% CI: 0.03–0.07). The mean osteotomy depth error (ODE) was 0.37 mm (95% CI: 0.15–1.35). Overpenetration was a characteristic finding, which indicates the necessity of a stop-trephine.

**Conclusions:**

Within the limitations of this study, we conclude that our results support the use of guided trephination for root-end resection.

## Background

The success rates of endodontic surgery, including root-end resection, are extremely variable. According to the literature, the range spans from 17 to 96% [1–3]. This extreme variability can be put down to the technique-sensitivity of these surgical interventions. However, the results of a meta-analysis suggested that the use of high-power magnification alone can elevate the success rate of endodontic microsurgical interventions to as high as 94% [1]. Similarly, ultrasonic retrograde preparation with modern root-end filling materials, such as MTA (mineral trioxide aggregate) and bioceramics is superior to shallow cavity preparation with adhesive materials in terms of healing [4].

All these innovations have been introduced to endodontic surgery in the last few decades, allowing more predictable outcomes. However, the operator factor remains unresolved, and this is a considerable source of error both in root-end resection and the osteotomy that precedes it. The challenge here is to direct the osteotomy in a way that allows the removal of the desired section as accurately as possible [5] - a goal that is extremely difficult to achieve solely via mental navigation. For this reason, cone-beam computed tomography (CBCT) is considered to be essential before periapical surgical procedures [6]. Indeed, CBCT is a great help, but the accuracy of the procedure still depends on how accurately the surgeon can mentally register the three-dimensional image with the actual clinical appearance of the corresponding structures. This leaves plenty of room for error, and the profession has been on the search for further and better navigation aids for some time.

In 2007, Pinsky et al. were the first to report on the computer-assisted design and manufacturing of surgical templates for endodontic application [7]. Comparing the guided approach to freehand surgery, they found the former to be significantly superior. The recent years have seen a renewed interest in surgical guides (templates) for endodontic surgery, possibly because stereolithographic manufacturing (i.e. 3D printing) has become widely available and development in this direction has become a real possibility [8–10]. Patel et al., in a case report, described the use of a 3D printed custom retractor for endodontic surgery [11]. Strbac and colleagues published a case report, where a stereolithographically fabricated surgical template was used to help the osteotomy and the root resections [12]. In these cases, though, the templates were not used to guide the osteotomy itself, as is usual in dental implantology.

It is becoming widely accepted that the placement of dental implants is more predictable and accurate when using 3D printed surgical templates [13]. Studies have shown that implant placement through a guide allowed a more accurate implementation of the virtual plan to the surgical site than freehand insertion [14]. Based on the results of their randomized controlled trial, Younes and colleagues suggest that fully guided surgery (i.e. all osteotomies and implant placement through guide) should be considered the gold standard approach instead of freehand surgery in dental implantation [15]. The application of this idea to endodontic surgery was, in fact, a logical next step.

The first case that can be considered a truly guided endodontic surgery case was described in 2018, by Giacomino et al. [16]. The authors concluded that guided endodontic microsurgery is useful for osteotomy and root-end resection when precise control of depth, diameter, and angulation of osteotomy are necessary. Further case reports followed. Ye et al. operated a left maxillary lateral incisor and canine with a 3D printed model to help the localization of the apices [17]. Ahn et al. used a surgical template to localize the apices in a mandibular molar with a thick buccal bone plate [18]. Interestingly, the authors used the template according to what implantology would call “the pilot protocol”- that is, only the initial osteotomy (“pilot” osteotomy) was performed with the help of the template, the rest of the procedure was done without it.

All in all, template-based guided surgery is becoming recognized as an option for endodontic surgery, but still, there is a relative scarcity of studies on this subject.

To test the validity of these observations, we carried out a prospective exploratory study in 2018–2019 in 11 patients. In the study, we resected 14 root ends with our template-and-trephine method, utilizing tooth-supported, stereolithographically fabricated surgical templates. The aim of the study was to give an approximation of the clinical safety and accuracy of this method. We hypothesized that a) intra- and postoperative complications would be no more frequent and severe than what is usual in freehand cases; b) the method would allow the resection of the root with the trephine in all cases, so no further manipulation to this end would be necessary; c) by utilizing this method, the vertical error of root-end resection and the error of osteotomy depth would not be greater than ±1 mm; and c) the angular accuracy of the osteotomies would be close to that of template-guided dental implantation.

## Materials and methods

### Patients

Eleven patients were enrolled (mean age: 48.9 ± 12.4 years). Seven of these patients were women (mean age: 45.4 ± 11.8 years), and four were men (mean age: 55.0 ± 11.0 years). The demographic and baseline clinical characteristics of the study population are given in Table 1. Lesion sizes - to give an approximation of the severity of the periapical process - were calculated as recommended by Kim et al. [19]: the mesiodistal (width), buccolingual (depth) and apicocoronal (height) dimensions were measured. All patients were referred for endodontic surgery by general dental practitioners to the Department of Operative and Esthetic Dentistry, Faculty of Dentistry, University of Szeged (Szeged, Hungary). Only front teeth were included with healthy periodontium and restored clinical crown. The inclusion criteria were persisting periapical lesion and pain with or without swelling, impossible or previously failed root canal revision, age between 18 and 75 years, and signed informed consent. Relative and absolute contraindications of endodontic surgery counted as exclusion criteria, as well as any other condition that would have put the patient at unacceptable risk during or after surgery. Patients with non-restorable clinical crown or damaged periodontium were also excluded. The study conformed to the Declaration of Helsinki “Ethical Principles for Medical Research Involving ‘Human Subjects”, adopted by the 18th World Medical Assembly, Helsinki, Finland, June 1964, as amended by the 64th World Medical Assembly, Fortaleza, Brazil, October 2013. Furthermore, the study observed the principles of Good Clinical Practice. The protocol was approved by the National Institute of Pharmacy and Nutrition of Hungary (Approval No. OGYÉI/43796–6/2018).

**Table 1:** Demographic and baseline clinical characteristics of the study population

Case	Patient	Sex	Age	Tooth	Lesion size (mm) width x height x depth	Swelling	Fistula
1	1	F	29	22	3.42 × 2.74 × 3.13	–	+
2	2	F	32	12	3.06 × 3.58 × 2.94	–	–
3	3	F	48	11	3.02 × 2.83 × 5.24	+	–
4	3	F	48	21	6.37 × 6.46 × 5.47	+	–
5	4	M	40	11	16.33 × 12.48 × 10.08	–	+
6	5	M	49	11	5.13 × 4.46 × 4.18	–	–
7	6	F	47	12	3.30 × 4.66 × 3.23	–	+
8	7	F	52	22	4.51 × 3.23 × 4.18	–	–
9	8	M	64	44	4.91 × 7.61 × 5.88	+	–
10	9	M	67	34	2.40 × 4.30 × 2.42	+	–
11	10	F	43	14	3.60 × 3.90 × 4.51	+	–
12	11	F	67	11	4.37 × 2.43 × 5.54	–	–
13	11	F	67	22	3.69 × 4.59 × 3.18	–	–

Thirteen teeth were treated in 11 patients, resulting in altogether 14 root end removals. The lesion sizes were calculated utilizing CBCT scans, as proposed by Kim et al. [16]: the maximum diameter of the lesions was measured in 3 directions parallel to the standardized axes: mesiodistal (L_x_), apico-coronal (Ly) and buccolingual (Lz)

### Presurgical procedures

Cone beam computed tomographies were acquired (iCAT Next Generation, Imaging Sciences-Kavo, Hatfield, PA, USA) with standard settings for all patients (120 kV, 5 mA, 9 s, voxel size: 250 μm, FOV: 110 mm; all scans in this study were done with these specifications). A bite block was used to ensure non-occlusion and a correct head position. A silicone impression (Zetaplus, Zhermack, Italy) was taken in a plastic tray (Hi-Tray, Zhermack, Italy), and it was scanned separately. The acquisition was always performed by an experienced radiologist according to the recommendations of the guide manufacturer (dicomLAB Dental, Ltd., Szeged, Hungary), with the minimum exposure necessary for adequate image quality [20].

**Fig. 1:**
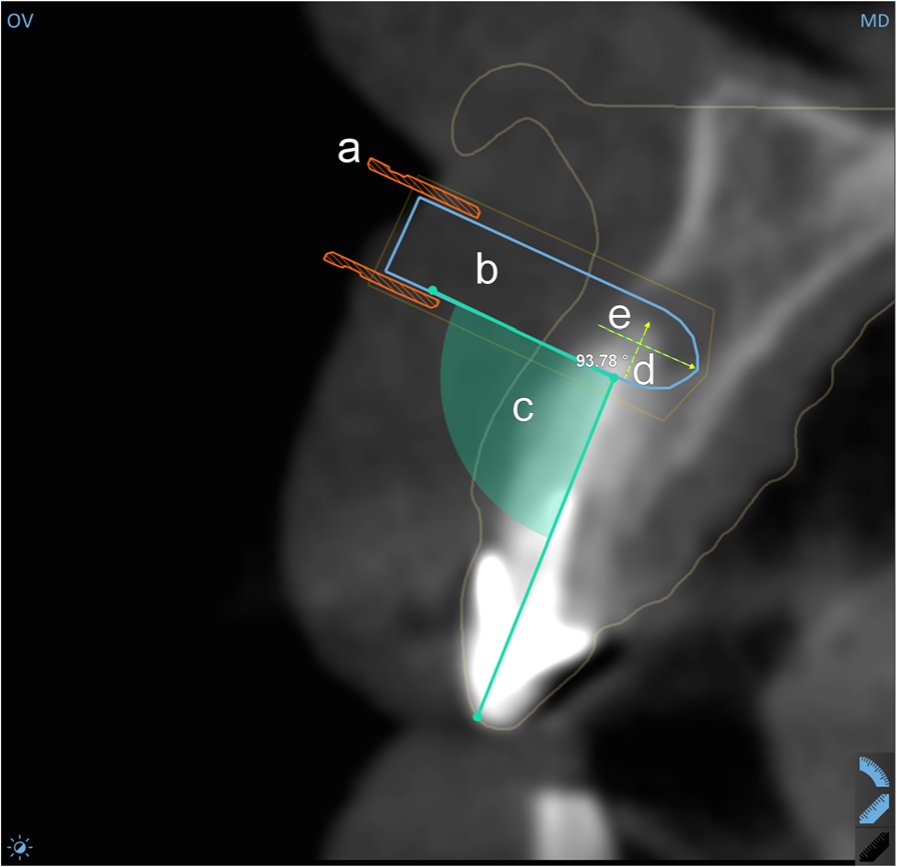
Surgical plan in the planning software (orovestibular view). **a** guiding sleeve; **b** virtual model to represent trephine; **c** the angulation of the planned osteotomy; **d** the planned depth of the osteotomy; **e** the planned length of the piece to be resected

**Fig. 2:**
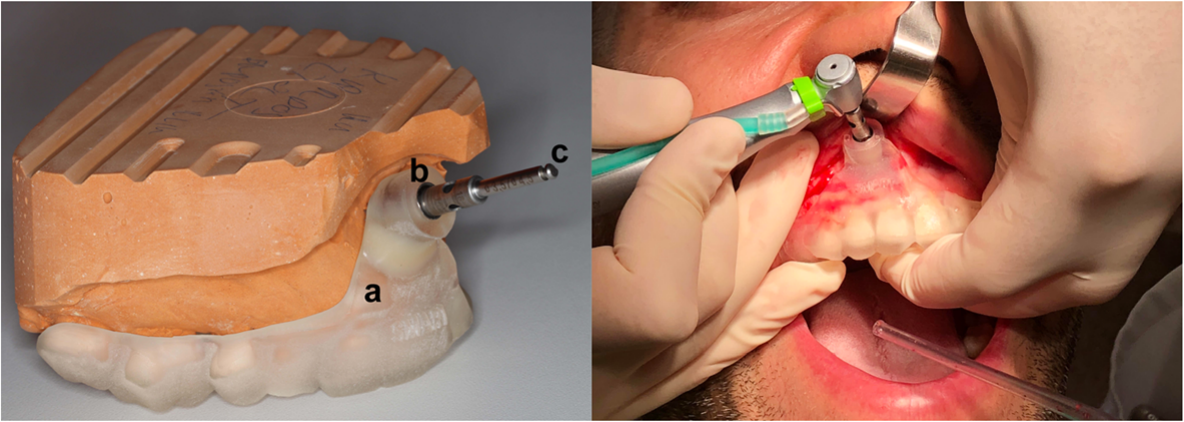
Left: the surgical setup demonstrated on a gypsum cast. **a** surgical template **b** guiding tunnel with metal sleeve; **c** trephine. Right: intraoperative image

The images were reconstructed as a volume (i-Cat Vision, Imaging Sciences International, Hatfield, PA, USA), and saved as DICOM files to provide input for surgical planning. The two scans were sent online to the template manufacturer, where they were registered, and sent back to the surgeon for planning. For 3D surgical planning, SMARTGuide 1.25 (dicomLAB Dental, Ltd., Szeged, Hungary) was used. For the planning of the surgeries, a virtual cylinder of the same dimensions as the actual trephine was used (Fig. 1). The only difference between the model and the trephine was that the model was rounded at the distal end, but this did not confound apical deviation calculations, as the axial lengths were the same, and for the calculations, two properly aligned models were compared (see below). This cylindrical model was positioned in a way that its axis was perpendicular to the tooth axis. The planned drilling length was 20 mm from the outer margin of the guiding sleeve in all cases. The surgical plans were prepared with the intention to resect 3 mm of the apical portion of the root. In cases with previous apicoectomy in history, only 1.3 mm was planned to be resected, always keeping in mind that enough root surface should be left to provide sufficient retention. All planning was performed by the same experienced surgeon, familiar with both implant and endodontic surgeries. The surgical templates were fabricated according to these plans, using a 3D printer (3D Systems ProJet MD 3510, USA). As a final step, to enhance the fit of the trephine in the guide, metal guiding sleeves of an inner diameter of 4.25 mm were inserted into the guiding tunnels of the templates. This diameter was wide enough to allow the rotation of the trephine of 4.21 mm outer diameter (see below) but narrow enough to allow only negligible lateral deviation. After fabrication, the templates were tried on the patients’ plaster casts to check correct and reproducible fitting (Fig. 2). A final check was performed right before each surgery, on the patients’ dentition. The insertion of the templates into the patients’ oral cavity was always preceded by disinfection, as per the manufacturer’s instructions.

### Surgical procedure

The surgeries were performed under local anesthesia (Ultracain D-S Forte 1:100000; Sanofi-Aventis GmbH). Full-thickness flaps were raised, the size and shape were always determined by the anatomical properties and accessibility of the current case.

As the surgical guide was placed, it defined the exact osteotomy site and the angle at which the osteotomy would be performed. For the osteotomy, a bone trephine was used with an outer diameter of 4.21 mm (Hager & Meisinger, Neuss, Germany) under copious irrigation. The trephine was applied through the guiding sleeve of the template (Fig. 2). 

After the combined osteotomy and apicoectomy, periapical curettage was performed if necessary, and retrograde preparation was performed using a piezosurgery unit (Piezomed, W&H, Austria). For the retrograde filling, MTA was used (Cerkamed, Poland). We applied methylene blue to visualize the ramifications. Before retrograde fillings, ferric-sulfate was used to ensure a bloodless working area. The retrograde preparation and filling were performed under high-power magnification (Opmi Pico, Zeiss, Germany). The flaps were closed and sutured with 5.0 monofilament sutures (Ethicon, USA). Within a month after the surgeries, a follow-up CBCT scan was made, with the same unit and settings as before the surgery. The sutures were removed 7 ± 1 days following the surgery, and follow-ups were scheduled at 6 months and 12 months.

### Analyses

The frequency and severity of intraoperative and postoperative complications were recorded, as well as the frequency of osteotomies when the root end was resected in the same step (i.e. no further manipulation was necessary for this purpose). Frequencies and percentages were calculated.

**Fig. 3:**
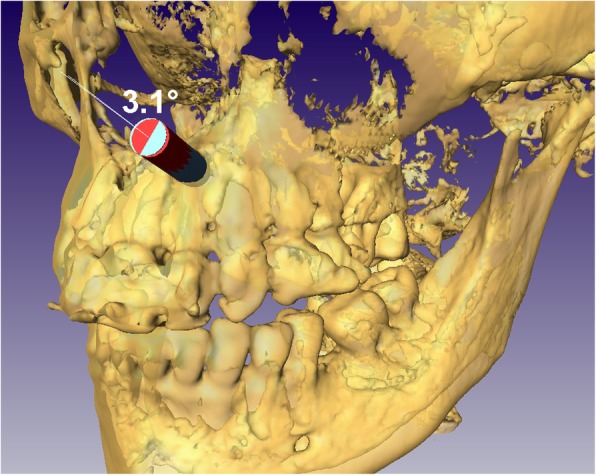
Analysis of angular deviation in Amira (blue: planned, red: realized). This figure does not depict the analysis of any of the actual cases, it is for illustration purposes only

The angular deviation was analyzed in Amira 5.4.0 (Thermo Fisher Scientific, USA) with dedicated algorithms. Pre- and postoperative CBCT scans of the given patient were transformed into the same coordinate system. For this registration, the region of interest was narrowed down to the analyzed bone (i.e. maxilla or mandible) to avoid inaccuracy stemming from differences in mouth opening. The bony tunnel formed by the trephine was manually segmented via a slice-by-slice method and transformed into a three-dimensional virtual model. As a next step, the cylindrical model used for planning was aligned with the model of the actual tunnel along their principal axes. The corresponding surgical plan was then extracted from the database of the planning and manufacturing system of the surgical guide and applied to a copy of the same cylindrical model. This way, it became possible to compare the result of the osteotomy with the plan in terms of the deviation of the principal axes (Fig. 3). The angular deviation was defined as the angle closed by the principal axes of the aligned models in degrees. The procedure was repeated three times for each case, and the mean of the three measurements was used for further analyses. The results were calculated as median (95% CI) as we found it more meaningful and informative in such a small sample than the usual mean (SD).

**Fig. 4:**
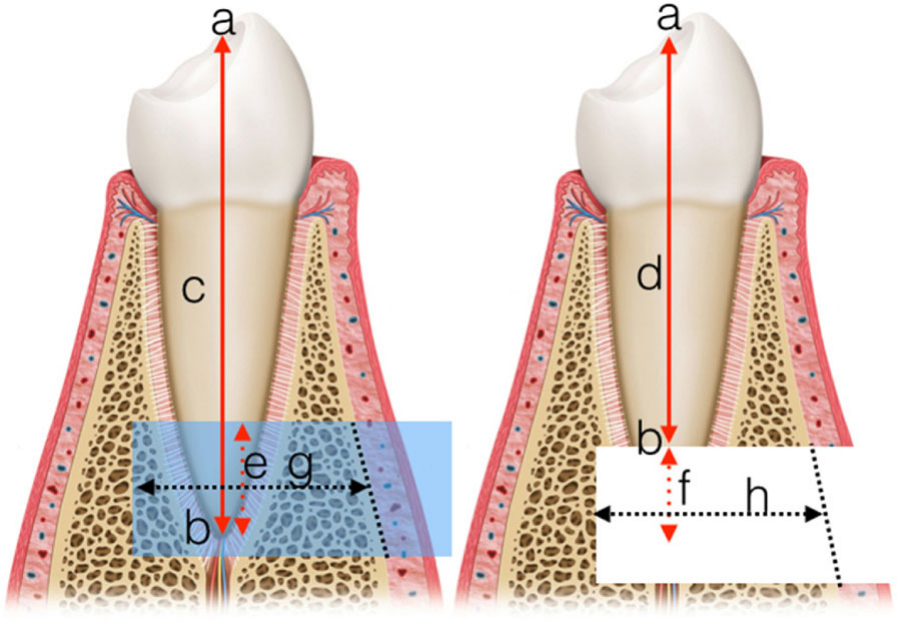
Explanation of the 2D measurements. Left: preoperative, Right: postoperative; **a**: coronal reference point; **b**: apical reference point (end points of the axis); **c**: axial length before surgery **d**: axial length after surgery; **e**: planned length of removal; **f**: actual resected length; **g**: planned depth of osteotomy; **h**: actual depth of osteotomy (for the measurements, the missing cortical was substituted by a straight line connecting the remaining cortical edges). Calculations: ARE = **e-f**; ODE = **g-h**

The length of the given tooth was measured in both the preoperative and postoperative CBCT images (i-CAT Vision, i-CAT, USA). This allowed the calculation of the length of the resected piece, which was subtracted from the planned length to be resected and so apex resection error (ARE) was calculated. Osteotomy depth error (ODE) was calculated similarly, by subtracting the actual depth from the planned depth (Fig. 4). The calculations were performed three times for each case, and the mean of the three measurements was used for further analyses. The results were calculated as median (95% CI), for the same reasons as given above.

All statistical calculations were done in SPSS 23.0 (IBM, USA).

## Results

**Fig. 5:**
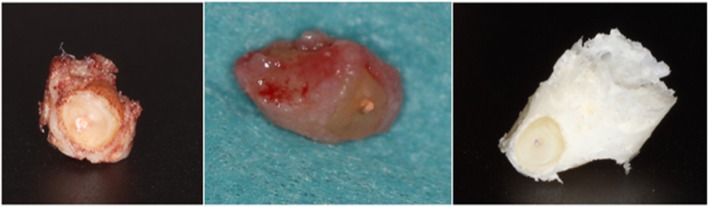
Bone cylinders removed with the trephine containing the resected root ends

**Fig. 6:**
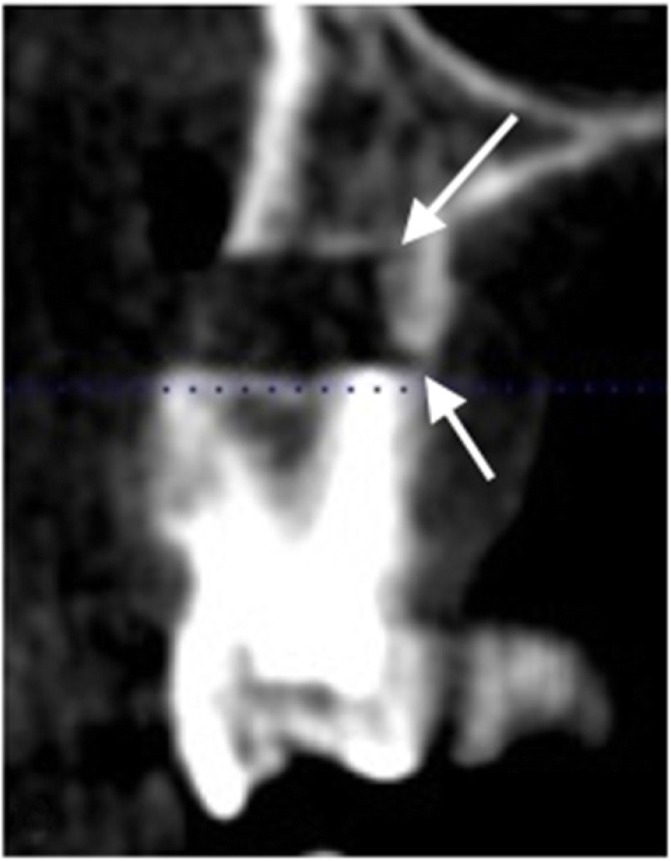
Overpenetration: note the trephine markings in the palatinal cortical (arrows)

Thirteen teeth were treated in 11 patients, resulting in altogether 14 apicoectomies. The root end was successfully and completely resected by the trephine in all cases, and in 11 cases, the resected piece was also removed with the trephine (Fig. 5). In 3 cases, the root end was removed with a periotome. No intraoperative complications were observed in any of the cases. In all cases, the surgery resolved the preoperative swelling and pain, and the patients were free of symptoms indicating recurrence or complications at the 6-month follow-up. In two of the cases, a key-hole extension of the trephine tunnel had to be performed in order to allow enough vertical space for the piezo tip for the retrograde preparation. In three cases, due to the extensive lesion and excochleation during the operation, the digital segmentation of the cavity was not possible as the borders could not be properly defined. Accordingly, in these cases, the angular deviation was not calculated. Some postoperative radiographs showed overpenetration (Fig. 6). The median angular deviation was 3.95° (95% CI: 2.1–5.9) (Table 2). The median apex removal error in the vertical plane (ARE) was 0.19 mm (95% CI: 0.03–0.07). The highest overcut was 0.93 mm, and the shortest cut fell behind the plan by 0.94 mm. In one case, exactly the planned length was cut. In 10 cases (71.4%), a longer piece of the apex was cut than planned, by a median of 0.37 mm (95% CI: 0.06–0.76). In 4 cases, the resected piece was shorter, by a median of 0.19 mm (*n* < 5, 95% CI not possible). The median osteotomy depth error (ODE) was 0.37 mm (95% CI: 0.15–1.35). Of the 13 remaining depth values, 9 (69.2%) indicated shallower osteotomy than planned by a mean of 0.71 mm, while the rest of the osteotomies were deeper than planned, by a mean of 0.31 mm. The highest overpenetration was 0.51 mm, while the shallowest penetration fell behind the plan by 1.56 mm.

**Table 2:** Results of the measurements

Case	Tooth	AD	ARE	ODE
1	22	NA	−.033	+ 0.29
2	12	4.0	−0.08	+ 1.35
3	11	6.3	−0.70	+ 0.13
4	21	2.1	− 0.76	− 0.15
5	11	NA	− 0.29	+ 0.56
6	11	3.9	−0.06	− 0.06
7	12	5.3	−0.03	+ 1.43
8	22	4.0	+ 0.15	+ 0.57
9	44	NA	−0.41	+ 1.56
10	34	2.4	0.00	−0.53
11	14^a^	1.5	−0.93	+ 0.23
11	14^a^	1.5	−0.15	+ 0.23
12	11	2.9	+ 0.94	+ 0.23
13	22	5.9	+ 0.23	−0.51
median		3.95	0.19	0.37
95%CI		2.10–5.90	0.03–0.70	0.15–1.35
min-max		2.10–6.30	−0.93-0.94	−0.51-1.56

*AD* angular deviation (degrees), *ARE* apex resection error (mm; -: longer than planned; +: shorter than planned), *ODE* osteotomy depth error (mm; -: deeper than planned; +: shallower than planned). In Case #11, two roots of the same tooth were treated. NA: in these cases, the given parameter could not be measured (see Results). ^a^Two roots of the same tooth were treated. Medians and corresponding confidence intervals were calculated from absolute values to express the degree of error regardless of its direction

## Discussion

Research in endodontic microsurgery unequivocally suggests that modern microsurgical approaches yield much higher success rates than traditional ones [21, 22]. Despite this, relatively little has been said in the literature about the use of such modern methods for root end localization and resection. The few available studies agree that guided root-end resection is efficient and more accurate than freehand surgery [23, 24]. To our knowledge, we are the first to report a series of clinical cases where osteotomy and root-end resection were carried out at the same time, with the same instrument (a bone trephine), using 3D printed surgical guides.

Our first hypothesis was that intra- and postoperative complications would be no more frequent and severe with the studied method than what is usual in freehand cases as reported by the literature and as shown by our own clinical experiencee. As we observed no complications at all during surgeries, we consider this hypothesis confirmed.

The second hypothesis was that the method would allow the resection of the root with the trephine in all cases, so no further manipulation to this end would be necessary. This hypothesis was also confirmed; the root end was successfully resected in all cases, and in most of the cases it was also removed with the trephine. This is practically important because this way the root end resection and removal procedure can be carried out in one simple step.

Our third hypothesis was that by utilizing this method, the vertical error of root-end resection and the error of osteotomy depth would not be greater than ±1 mm. This hypothesis was only partially confirmed, regarding the error of root end resection, which remained within 1 mm in both directions in the vertical plane (− 0.93 mm to + 0.94 mm). This indicates that the guidance was quite efficient (as reflected also by the angular deviation measurements, see below). The error of the depth of the osteotomy, however, exceeded the ±1 mm limit in three instances, which indicates that the method was less accurate in the horizontal dimension. These deviations indicated underpenetration (the fact that this did not affect the success of resection indicates that the planned depth was excessive in these cases). At the same time, overpenetration was also a recurrent finding, even if within the ±1 mm limit (Fig. 6). What these results suggest in general is that repeated depth check with a periodontal probe and observing the markings on the trephine are not enough to make the surgeon confident about this dimension. We propose that trephines with a stop (like implant drill bits) could resolve this problem.

Our last hypothesis was that the angular accuracy of the osteotomies would be close to that of template-guided dental implantation. We formulated this hypothesis like this because ours is the first study to assess this parameter for guided trephination, so we had nothing else to compare our results against. At the same time, this is a very important parameter, as it can determine if the root-end resection is successful (i.e. if a large enough part is resected to get rid of all the accessory canals, the very aim of the procedure). This last hypothesis was also confirmed. Tahmaseb et al., in their meta-analysis on guided implantation with tooth-supported guides, reported an overall angular deviation of 3.5° (studies of full and partial edentulousness included) [25]. Endodontic surgical guides cannot be considered entirely tooth-supported guides, Of course, it is the teeth that the template rests on, but the direction of the operation is not perpendicular to the occlusal plane, which would help to keep the guide in place. Rather, the operation happens perpendicular to the soft tissue, which adds instability to the system. The poorer accuracy of mucosa-supported guides is a known problem in guided implantology [26]. Following from these, one would expect the angular deviation to be slightly poorer than but still comparable to that of implant guides. The median deviation of 3.95° we found confirms that expectation.

Beyond addressing our hypotheses, we would like to point out a practical difficulty we often faced. Obviously, to have the guiding sleeve at the level of the apex, the impression (that serves as the model for the template) must contain information on the tissues at that level. Otherwise the planned sleeve falls outside the template, and the guide cannot be produced. In other words, the achievable depth of the impression is a limitation of digital planning, and this is probably true for digital impressions as well. Although we used orthodontic impression trays, in some cases the impressions had to be retaken as they were not deep enough. Sometimes this did not help either. In those cases, a compromise had to be made, and the trephination was planned not exactly at the originally intended 90°. That is, the planned trephination path was not exactly perpendicular to the axis of the tooth. Naturally, such a compromise is allowable only if it does not risk the aim of the surgery, and the decision requires careful consideration. Another aspect of the same problem is sometimes the patient’s lip had to be retracted almost to an extreme degree to allow access with the trephine through the guide in the proper direction.

We find that our study offers valuable insight into the accuracy and clinical characteristics of the studied method, but we must mention two limitations that have to be kept in mind when interpreting the results.

First, as our study is the first of its kind, no comparison with the literature is possible. While there is a multitude of accuracy studies for guided dental implant surgery, the interest in guided root-end resection is quite new, and research into this topic is in the exploratory phase. Still, we consider it important to share our experience, as the literature shows that the method is becoming widespread. Our data indicate that the method is safe and accurate enough for the purpose.

Second, while there is an established methodology for the assessment of accuracy of dental implantation, including the assessment of coronal and apical deviations and various other measures, there is no such recognized methodology for the assessment of trephination. Therefore, we had no option but to choose our measures ad hoc. Of the methods of measurement we had access to, we chose the ones we considered to be the clinically most relevant. This is, however, not to say that the measures presented here are the best to characterize trephination accuracy.

## Conclusions

We conclude that our results support the use of guided trephination for root-end resection. However, the research is in an early stage, and there is ample room for the improvement of both the method and its assessment.

## Data Availability

The datasets used and/or analysed during the current study are available from the corresponding author on reasonable request.

## Abbreviations

ARE: Apex removal error
CBCT: Cone Beam Computed Tomography
CI: Confidence Interval
DICOM: Digital Imaging and Communication in Medicine
FOV: Field of view
MTA: Mineral trioxide aggregate
ODE: Osteotomy depth error
